# Association between Inflammatory Conditions and Alzheimer’s Disease Age of Onset in Down Syndrome

**DOI:** 10.3390/jcm10143116

**Published:** 2021-07-15

**Authors:** Florence Lai, Nathaniel Mercaldo, Cassandra M. Wang, Giovi G. Hersch, Herminia Diana Rosas

**Affiliations:** 1 Department of Neurology, Harvard Medical School, Massachusetts General Hospital, 149 13th Street, Charlestown, MA 02129, USA; rosas@helix.mgh.harvard.edu; 2 Department of Radiology, Center for Neuroimaging of Aging and Neurodegenerative Diseases, Athinoula A. Martinos Center for Biomedical Imaging, 149 13th Street, Charlestown, MA 02129, USA; nmercaldo@mgh.harvard.edu; 3 Harvard College, Harvard University, Cambridge, MA 02130, USA; cassandrawang@college.harvard.edu; 4 College of Arts and Sciences, Boston University, Boston, MA 02215, USA; herschgi@bu.edu

**Keywords:** Down syndrome, medical co-morbidities, inflammatory conditions, Alzheimer’s disease

## Abstract

Adults with Down syndrome (DS) have an exceptionally high prevalence of Alzheimer disease (AD), with an earlier age of onset compared with the neurotypical population. In addition to beta amyloid, immunological processes involved in neuroinflammation and in peripheral inflammatory/autoimmune conditions are thought to play important roles in the pathophysiology of AD. Individuals with DS also have a high prevalence of autoimmune/inflammatory conditions which may contribute to an increased risk of early AD onset, but this has not been studied. Given the wide range in the age of AD onset in those with DS, we sought to evaluate the relationship between the presence of inflammatory conditions and the age of AD onset. We performed a retrospective study on 339 adults with DS, 125 who were cognitively stable (CS) and 214 with a diagnosis of AD. Data were available for six autoimmune conditions (alopecia, celiac disease, hypothyroidism, psoriasis, diabetes and vitamin B12 deficiency) and for one inflammatory condition, gout. Gout was associated with a significant delay in the age of AD onset by more than 2.5 years. Our data suggests that inflammatory conditions may play a role in the age of AD onset in DS. Further studies are warranted.

## 1. Introduction

The amyloid cascade hypothesis [1] has been the dominant pathogenic explanation of Alzheimer disease (AD) in Down syndrome (DS), which occurs in upward of three-quarters of older adults with DS [2,3,4]. This is in part due to the overexpression of amyloid precursor protein (APP), which is triplicated on chromosome 21 and results in an excess deposition of brain amyloid as early as the teenage years [5,6]. Although the typical age of onset of AD in people with DS is approximately twenty years earlier than in those with sporadic AD [7], there is considerable heterogeneity in the age of onset of AD in DS, with some individuals developing AD at as young as 40 and some after the age of 70 [7]. Factors which underlie this considerable variability are still unknown but may provide important insights.

Over the past few decades, immunologic processes, neuroinflammation and oxidative stress have become recognized as playing prominent roles in AD in the general population [8,9,10,11]. In the brain, microglia are activated by the presence of β amyloid and molecules from degenerating neurons. They secrete proinflammatory cytokines such as IL1, IL6, tumor-necrosis-factor-α (TNF-α) and reactive oxygen species (ROS) [12]. Similarly, peripheral immune processes have been demonstrated to also contribute to AD and its progression [13]. Autoimmune conditions involve peripheral immunological inflammatory processes that result from a complex interplay of genetic polymorphisms and/or environmental factors; these factors may provide an increased risk for dementia [14].

Similar complex processes may also be at play in adults with DS. Several genes located on chromosome 21 are involved in inflammation and in autoimmune regulation and are related to oxidative stress [15,16,17,18]. It has been recognized that autoimmune conditions—including those relating to the thyroid, celiac disease, alopecia areata or universalis, type 1 diabetes, psoriasis, and Vitamin B12 deficiency—are more prevalent in individuals with DS than in the population at large [19,20,21,22,23,24]. It is also possible that these same processes may contribute to the early and variable age of onset of AD in DS. In this study, we sought to determine the association of inflammatory/autoimmune medical comorbidities with the age of AD onset in a large retrospective study of adults with DS.

## 2. Materials and Methods

We performed a retrospective study of medical records from January 2008–June 2020, including a comprehensive medical history and neuropsychological assessments of 339 adults with DS over the age of 40 followed annually in a DS neurology specialty clinic, to assess the association between the age of AD onset and presence of inflammatory and/or autoimmune conditions. Follow-up ranged from two to 13 years. Data were available for the following medical comorbidities: hypothyroidism, celiac disease, diabetes (including Type 1 and Type 2), alopecia areata/universalis, psoriasis, Vitamin B12 deficiency and gout.

### 2.1. Clinical Assessments

The cognitive status was determined by a neurologist with expertise in the assessment of adults with DS with dementia in a manner generally consistent with the AAMR-IASSID Working Group for the Establishment of Criteria for the Diagnosis of Dementia in Individuals with Developmental Disability [25]. A diagnosis of AD was given when a patient demonstrated more than one year of progressive and relentless decline in two or more areas of cognition and function and/or behavior that was not otherwise explained. The age of AD onset most closely approximates the age of AD symptom onset in this prospectively followed cohort. The cohort was divided into those who were cognitively stable (CS) and those with possible or probable Alzheimer disease (AD). The study focused on identifying inflammatory conditions that we hypothesized could influence the age of AD onset. The most recent visit was used both for the diagnostic classification and identification of medical comorbidities.

### 2.2. Statistical Analyses

Descriptive summaries were computed for the entire cohort and by diagnosis (CS and AD). Continuous variables were summarized as the median and IQR (interquartile range; 25–75 percentiles) and categorical variables as frequencies (proportions). Differences in the distributions of categorical and continuous variables by diagnosis were assessed using either the chi-square test/Fisher’s exact test or the Mann–Whitney test, respectively.

Separate linear regression models were constructed to quantify the association between the age of AD onset and each autoimmune condition, history of gout, and ApoE-ε4 status. Parameter estimates from these models, and their 95% confidence intervals, were computed to summarize expected differences of the age of AD onset by autoimmune condition (e.g., alopecia vs. no alopecia), history of gout, and ApoE ε4 status. Due to the rarity of many of the autoimmune conditions, simultaneous adjustments for ApoE ε4 status were not performed when characterizing the relationship between the age of onset and the presence of each autoimmune condition. Given that these variables were unrelated to autoimmune diseases, the resultant parameter estimates remain unbiased [26].

## 3. Results

### 3.1. Demographics

The demographics for the entire cohort and by diagnosis are presented in Table 1. The cohort consisted of 125 individuals (37%) who were cognitively stable (CS) and 214 individuals (63%) with possible or probable Alzheimer disease (AD). As expected, the CS cohort was younger than the AD cohort (median: 51 vs. 57 years of age; *p* < 0.001). There was insufficient evidence to conclude differences between groups with respect to sex or the presence of an ApoE ε4 (2/4; 3/4; 4/4) genotype. The average age of AD onset in the AD group was 53 years [interquartile range 48, 57].

### 3.2. Inflammatory/Autoimmune Condition by Cognitive Status

The frequency of each inflammatory/autoimmune condition is provided in Table 2. Approximately 78% of the study cohort had at least one autoimmune condition; 22% had none. Hypothyroidism was the most frequent inflammatory condition (63%), but we were unable to detect differences in the frequency of hypothyroidism between the CS and AD cohorts. The proportion of subjects with diabetes (9.6% vs. 3.7%, *p* = 0.049) was greater among the CS subjects compared to the AD subjects. Differences between the cohorts with respect to the presence of autoimmune alopecia (*p* = 0.86), celiac disease (*p* = 0.31), psoriasis (*p* = 0.06), Vitamin B12 deficiency (*p* = 0.12) or gout (*p* = 0.20) were not detected.

We also evaluated the total number of inflammatory conditions to determine if the risk for AD might be associated with the total number of conditions. As shown in Table 3, there was a marginally significant relationship between the cumulative number of inflammatory conditions between the CS and AD groups. Interestingly, 51.6% of the total sample had one condition, 20.1% had two, and more than 6% had three or more conditions.

### 3.3. Association between Age of AD Onset and Inflammatory/Autoimmune Conditions

We also quantified the association between any of the inflammatory/autoimmune conditions and the age of onset of dementia. As shown in Table 4, patients with a history of gout had, on average, an age of AD onset 2.58 (95% CI: 0.09–5.07) years later than those without gout (*p* = 0.043). We did not detect any effect of gender on the association between gout and the age of AD onset (*p* = 0.60). We were unable to detect an association between the age of AD onset and a diagnosis of any of the other inflammatory/autoimmune conditions.

In addition, a single regression model was also constructed in which the age of AD onset was regressed onto all autoimmune conditions simultaneously. The results, similarly, did not demonstrate an association between the number of autoimmune conditions and the age of onset of dementia (data not shown).

## 4. Discussion

Our study is the first to analyze the relationship between inflammatory and/or autoimmune medical comorbidities commonly present in DS and the age of AD onset. In this large retrospective study, we found that the presence of gout conferred a significant delay in the onset of AD in DS by more than two years. A potential “protective” effect of gout and hyperuricemia has been suggested in several neurodegenerative diseases such as Parkinson disease and sporadic AD [27,28], in which neuroinflammatory processes and oxidative stress have been found to play important roles [29]. The higher incidence of hyperuricemia in DS than in the neurotypical population [30] may be related to the presence of two genes involved in the regulation of purine synthesis on chromosome 21 [31] but could also represent a compensatory natural antioxidant response [28,32] to redox imbalances in DS [33] due in part to excessive levels of superoxide dismutase, which damages neurons and which is also on chromosome 21 [34]. Our findings may have implications for the potential use of antioxidant therapies for AD in DS, as any treatment that could delay AD onset in this population would be worthwhile. The potentially protective effect of gout for AD also raises an important point about the use of uric-acid-lowering medications, especially as at least one report indicates that low uric acid levels are associated with AD [35]. Perhaps a symptomatic treatment for gout with strong anti-inflammatory agents should be used instead. However, future studies need to evaluate these considerations.

We did not find any significant association between the age of onset of AD and the presence of any of the autoimmune conditions studied. This was true whether these autoimmune conditions were analyzed separately or together. This may not be entirely unexpected, given the mixed results in the general population regarding inflammatory comorbidities and may be especially true for thyroid conditions [36,37]. For example, some studies have suggested that thyroid hormone might regulate the expression of amyloid precursor protein [38,39], and autoimmune thyroid disease has been reported to occur with a very high prevalence in familial AD [40]; however, the mechanisms that could underlie this association are not entirely clear [36,41]. Likewise, studies in celiac disease have not found an association with the age of onset in sporadic AD [42]. Although a higher incidence of Vitamin B12 deficiency has been found in individuals with neurodegenerative disorders [43], no causative relationships have been described [44]. In contrast, several epidemiological studies have found a relationship between diabetes and the risk of AD [45] in the general population. Individuals with psoriasis have been reported to have an increased risk of developing dementia [46].

It is possible that the absence of an association between autoimmune conditions and the age of AD onset in our study reflects the relatively small sample size. More importantly, it is possible that the cumulative years of exposure to the immunological alterations in autoimmune conditions might be a more relevant risk factor [47] than the actual presence of the condition itself; however, data regarding the duration of the respective medical conditions was not available. It is also possible that the complex interplay of distinct immune processes, some of which could confer some “protection” and others a “higher risk”, may explain some of our results, especially as many of our cohort had more than one autoimmune condition. Our study suggests that future studies that include both the presence and duration of inflammatory medical comorbidities and their biomarkers in the DS population are warranted [48], considering the multiplicity of genes that affect immunity in DS.

In summary, our study suggests that anti-inflammatory strategies should be considered in the DS population and provides support for future studies focused on the potential influence of medical comorbidities on the age of onset of dementia in adults with Down syndrome.

## Figures and Tables

**Table 1:** Descriptive summaries by cognitive status. Continuous variables are summarized as median and interquartile ranges and categorical variables are summarized as frequencies and percentages N, (%).

	All *N* = 339	Cognitively Stable (CS) *N* = 125	Alzheimer Disease (AD) *N* = 214	*p*
Sex (Male)	197 (58.1)	78 (62.4)	119 (55.6)	0.267
Age	55 (50,60)	51 (46,56)	57 (53,62)	<0.001
ApoE ε4	66 (24.3)	21 (23.9)	45 (24.5)	1.000

**Table 2:** Frequency (percent) of inflammatory conditions by cognitive status. N (%).

Inflammatory/Auto-Immune Condition	All *N* = 339	CS *N* = 125	AD *N* = 214	*p*-Value
Alopecia areata or universalis	30 (8.8) *	12 (9.6)	18 (8.4)	0.862
Celiac disease	20 (5.9)	10 (8.0)	10 (4.7)	0.310
Hypothyroidism	212 (62.5)	75 (60.0)	137 (64.0)	0.534
Psoriasis	34 (10.0)	18 (14.4)	16 (7.5)	0.063
Diabetes	20 (5.9)	12 (9.6)	8 (3.7)	0.049 *
Vitamin B12 deficiency	59 (17.4)	16 (12.8)	43 (20.1)	0.119
Gout	45 (13.3)	21 (16.8)	24 (11.2)	0.195

* *p* ≤ 0.05 is significant.

**Table 3:** Cumulative number of inflammatory conditions by cognitive status, N (%).

Number of Inflammatory/ Autoimmune Conditions	All	CS	AD	*p*-Value
**0**	76 (22.4)	31 (24.8)	45 (21.0)	0.076
**1**	175 (51.6)	60 (48.0)	115 (53.7)	
**2**	68 (20.1)	23 (18.4)	45 (21.0)	
**3**	16 (4.7)	7 (5.6)	9 (4.2)	
**4**	4 (1.2)	4 (3.2)	0 (0.0)	

**Table 4:** Linear regression estimates summarizing the expected difference in the age of onset among those with and without each autoimmune/inflammatory condition.

Inflammatory/Autoimmune Condition	Estimate (95% CI)	*p*
Alopecia	2.36 (−0.48, 5.21)	0.103
Celiac disease	−1.12 (−4.88, 2.64)	0.559
Hypothyroidism	0.52 (−1.14, 2.17)	0.541
Psoriasis	−1.53 (−4.55, 1.48)	0.319
Diabetes	−1.08 (−5.27, 3.10)	0.612
Vitamin B12 deficiency	0.76 (−1.22, 2.74)	0.449
Gout	2.58 (0.09, 5.07)	0.043 *

* Significance is *p* ≤ 0.05.

## Data Availability

The data presented in this study are available upon request from the corresponding author. The data are not publicly available due to privacy restrictions.
